# The impact of nutritional risk factors and sarcopenia on survival in patients treated with pelvic exenteration for recurrent gynaecological malignancy: a retrospective cohort study

**DOI:** 10.1007/s00404-021-06273-7

**Published:** 2021-11-03

**Authors:** Veronika Seebacher, Andrea Rockall, Marielle Nobbenhuis, S. Aslam Sohaib, Thomas Knogler, Rosa M. Alvarez, Desiree Kolomainen, John H. Shepherd, Clare Shaw, Desmond P. Barton

**Affiliations:** 1 Department of Gynaecological Oncology, The Royal Marsden NHS Foundation Trust, 203 Fulham Road, Chelsea, London, SW3 6JJ UK; 2 Department of Gynaecology and Gynaecologic Oncology, Medical University of Vienna, Waehringer Guertel 18-20, 1090 Vienna, Austria; 3 Department of Radiology, The Royal Marsden NHS Foundation Trust, 203 Fulham Road, Chelsea, London, SW3 6JJ UK; 4 Department of Biomedical Imaging and Image-Guided Therapy, Medical University of Vienna, Waehringer Guertel 18-20, 1090 Vienna, Austria; 5 Department of Gynaecologial Oncology, King’s College Hospital NHS Foundation Trust, Denmark Hill, London, SE5 9RS UK; 6 Department of Nutrition and Dietetics, The Royal Marsden NHS Foundation Trust, Downs Road, Sutton, SM2 5PT UK

**Keywords:** Cachexia, Malnutrition, Muscle attenuation, Cervical cancer, Endometrial cancer, Vulvar cancer

## Abstract

**Purpose:**

The aim of the present study is to investigate the prognostic significance of nutritional risk factors and sarcopenia on the outcome of patients with recurrent gynaecological malignancies treated by pelvic exenteration.

**Methods:**

We retrospectively evaluated muscle body composite measurements based on pre-operative CT scans, nutritional risk factors as assessed by a validated pre-operative questionnaire, and clinical–pathological parameters in 65 consecutive patients with recurrent gynaecological malignancies, excluding ovarian cancer, treated by pelvic exenteration at the Royal Marsden Hospital London. Predictive value for postoperative morbidity was investigated by logistic regression analyses. Relevant parameters were included in uni- and multivariate survival analyses.

**Results:**

We found only (1) low muscle attenuation (MA)—an established factor for muscle depletion—and (2) moderate risk for malnutrition to be independently associated with shorter overall survival (*p* = *0.006 *and* p* = *0.008*, respectively). MA was significantly lower in overweight and obese patients (*p* = *0.04*). Muscle body composite measurements were not predictive for post-operative morbidity.

**Conclusion:**

The study suggests that pre-operative low MA and moderate risk for malnutrition are associated with shorter survival in patients with recurrent gynaecological malignancies treated with pelvic exenteration. Further studies are needed to validate these findings in larger cohorts.

**Supplementary Information:**

The online version contains supplementary material available at 10.1007/s00404-021-06273-7.

## Introduction

Pelvic exenteration is the last therapeutic option in selected, heavily pre-treated patients with pelvic recurrent or persistent gynaecological malignancies. Due to improvements in surgical techniques, perioperative management and patient selection, postoperative 5 year overall survival (OS) rates ranging from 20 to 73% can be achieved while postoperative mortality rates have decreased from 20% to less than 5% [1–8]. However, pelvic exenteration remains a morbid intervention with approximately 50% of patients suffering a major perioperative complication [3–6, 9, 10]. Therefore, patients are selected who are most likely to benefit and who are physically and psychologically fit enough to tolerate pelvic exenteration.

Over the past decade, cachexia, a multifactorial syndrome characterized by systemic inflammation and hypermetabolism, involuntary weight loss, and loss of skeletal muscle mass, and its impact on adverse clinical outcome in cancer patients has been extensively investigated [11–18]. Cachexia has been estimated to affect approximately 50% of all cancer patients and to account for up to 20% of cancer deaths [19].

One of its key components is sarcopenia, a syndrome characterised by progressive loss of skeletal muscle mass and strength. While sarcopenia is most commonly observed in older people, it can also develop secondary to prolonged immobility, inadequate dietary intake, malabsorption, inflammatory disease, or malignancy [20]. Both the presence of cancer and its treatment tend to disrupt homeostasis and lead to important metabolic changes to maintain homeostasis [19]. Computed tomography (CT) imaging does not directly measure cachexia. However, it can be used to estimate both the muscle mass and the muscle density. This is done by assessing the lumbar skeletal muscle area, by calculating the muscle attenuation (MA), a measure for muscle density, with lower values reflecting increased muscular lipid content [11, 18], and subsequently by calculating the skeletal muscle index (SMI). Both reduction of muscle mass and loss of muscle density have been associated with shorter survival in various malignancies [12–16], decreased tolerance to anti-cancer treatment [17, 21], and increased postoperative morbidity [22, 23].

The aim of the present study is to investigate the effect of nutritional risk factors, as assessed in a questionnaire, and body composition, i.e. skeletal muscle mass and MA assessed by CT imaging, on postoperative morbidity and survival in patients undergoing pelvic exenteration for recurrent or persistent gynaecological malignancies.

## Materials and methods

### Patients’ cohort and data acquisition

We included patients who underwent pelvic exenteration for recurrent or persistent gynaecological malignancy at the Gynaecological Oncology Unit of the Royal Marsden Hospital (RMH), London, between 2000 and 2015. Clinical, pathological and radiological data were extracted retrospectively from electronic and paper-based medical records. Patients were excluded from analyses if the primary tumour was of ovarian origin, and/or if surgical data were missing or incomplete (see supplementary Fig. 1).

### Clinical management

Prior to pelvic exenteration, clinical and radiological assessment was performed including an examination under anaesthesia, including diagnostic laparoscopy to evaluate operability [24]. Patients were only eligible for surgery if extrapelvic disease was excluded and if there was the expectation of achieving an R0 resection. We used the American Society of Anesthesiologists (ASA) physical status classification system and the age adjusted Charlson Comorbidity Index (AACCI) to assess performance status and perioperative risks, respectively [25, 26]. Postoperative morbidity and mortality were assessed within 60 days after pelvic exenteration and was classified according to the Clavien–Dindo classification of surgical complications [27]. We defined progression-free survival (PFS) as the date of first clinical or radiological evidence of progressive disease.

### Anthropometric measurements

Height (cm) and weight (kg) prior to pelvic exenteration were annotated in the majority of patients during pre-assessment. Since 2008, risk factors for malnutrition and weight history were assessed routinely using the Royal Marsden Nutrition Screening Tool (RMNST) [28]. The RMNST is a nutritional screening tool developed by the Department of Nutrition and Dietetics of the RMH for inpatient use. It has been designed and validated to assess the risk of malnutrition at any time point in an oncology inpatient setting. In addition to the assessment of weight loss and food intake habits, it incorporates parameters characteristically affecting cancer patients such as mucositis, nausea, vomiting and dysphagia. The questionnaire allows stratification of patients into three risk groups for malnutrition: “low risk” (score of 0–4), “medium risk” (score of 5–9), and “high risk” (score of 10 +) [27].

### CT image analysis

Only CT scans performed within 2 months prior to pelvic exenteration were used for analyses. CT scans were analysed by two independent researchers (VS and AR) trained in radiologic anatomy and body composition analysis, blinded to the outcome of the surgery. Two adjacent axial images within the same series, at the level of the third lumbar vertebra, were identified. Using predefined Hounsfield Unit (HU) ranges, the total cross-sectional areas (cm^2^) of skeletal muscle tissue (− 29 to 150 HU), including the rectus abdominis, transversus abdominis, internal and external obliques, psoas, quadratus lumborum, and erector spinae muscles, the visceral adipose tissue (VAT; − 150 to − 50 HU), and the subcutaneous adipose tissue (SAT; − 190 to − 30 HU) were determined and analysed using Slice-O-Matic software as previously described (v5.0, Tomovision, Montreal, Quebec, Canada) [11, 29]. In addition, the mean MA, was assessed by calculating the average HU value of the total muscle cross-sectional area. Values for cross-sectional surface of muscle (MS), visceral fat (VAT), subcutaneous fat (SAT), and muscle attenuation (MA) were averaged for each patient using two adjacent axial images within the same series at the level of the third lumbar vertebra. Subsequently, they were normalized for height in meters squared (m^2^) and reported as lumbar skeletal muscle index (SMI; cm^2^/m^2^), lumbar visceral adipose tissue index (VATI; cm^2^/m^2^) and subcutaneous adipose tissue index (SATI; cm^2^/m^2^), respectively.

### Statistical analysis

Values are given as medians (interquartile range [IQR]). Differences in patients’ characteristics were analysed using the Pearson Chi-Square Test. The Mann–Whitney *U* test was used to compare medians of MA and MS between risk groups of clinico-pathological parameters, i.e. age (< vs. ≥ the median age of 56 years), BMI (< vs. ≥ 25 kg/m^2^), and nutritional assessment according to the RMNST (low vs. moderate risk for malnutrition). We used 41.0 cm^2^/m^2^ as a cut off for SMI as previously described [11]. As the number of patients in the present study was too small to generate cut-off values by optimum stratification for MA, VATI and SATI, we used the quartiles of these values to generate risk groups. Cut-off values were set at the lowest quartile for MA, and at the highest quartile for VATI and SATI. Survival probabilities were calculated by the product limit method of Kaplan and Meier. Differences between groups were tested using the log-rank test. The results were analysed for the endpoints of progression-free survival and overall survival (i.e. time between pelvic exenteration and date of disease recurrence/progression or date of death due to all causes, respectively). Patients alive with no or stable disease were censored with the date of last follow-up. Uni- and multivariate Cox regression models were performed, comprising all variables that generated a *p* value of < 0.05 in univariate analysis.

Results of uni- and multivariate survival analyses are given as *p* value [hazard-ratio (HR)])and 95% confidence interval (95% CI). Logistic regression analyses were performed to assess the predictive value of MA, SMI, VATI, and SATI risk groups and other clinical-pathological parameters for severe 60 days postoperative morbidity (i.e. Clavien–Dindo grade ≥ 3). *p* values < 0.05 were considered statistically significant and all tests are two-sided. We used the statistical software SPSS 22.0 for Mac (SPSS 22.0.0, SPSS Inc., Chicago, IL) for statistical analysis.

## Results

### Patients’ characteristics

A total of 76 patients underwent pelvic exenteration for recurrent or persistent gynaecological malignancy at the RMH from 2000 to 2015. After exclusion of patients with ovarian cancer and those in whom essential data elements were missing, 65 patients could be included into the present study. A flowchart depicting the patient selection process is shown in supplementary Fig. 1 (S1). Indications for pelvic exenteration were recurrent/persistent cancer of the vulva/vagina in 25 (38.5%) patients, of the cervix uteri in 31 (47.7%), and of the uterus in 9 (13.8%). Histological subtypes of the primary cancer were adenocarcinoma in 17 patients (26.2%), squamous cell carcinoma in 40 (61.5%), clear cell carcinoma in 3 (4.6%), mixed mullerian tumour in 1 (1.5%), and sarcoma in 4 (6.2%). Fifty-six patients (86.2%) had radiotherapy in the past, 30 (46.2%) had 2 or more previous lines of treatment. No patient received parenteral nutrition (PN) pre-operatively. Patients’ characteristics at the time of pelvic exenteration are listed in Table 1.

**Table 1:** Characteristics and body composition measurements in 65 patients treated with pelvic exenteration for gynaecological malignancy

	*n* or median	% or IQR
Median age (IQR) in years	55	44–64
ECOG		
0–1	54	83.1
2–3	4	6.2
NA	7	10.7
ASA		
0–1	14	21.5
2–3	35	55.4
NA	15	23.1
AACCI		
2	19	29.2
3	13	20
4	10	15.4
5	13	20
6	4	6.2
7	1	1.5
NA	5	7.7
Sarcopenia measurements (*n* = 32)		
Median lumbar total muscle cross-sectional area (cm^2^)	113.8	104.9–129.7
Median SMI (cm^2^/m^2^)	43.6	40.1–50.9
SMI < 41 cm^2^/m^2^	8	25.0
Median MA (HU)	40.5	31.1–44.3
MA < 31 HU	7	20.6
Median VATI (cm^2^/m^2^)	87.4	67.5–120.9
VATI > 120 cm^2^/m^2^	8	23.5
Median SATI (cm^2^/m^2^)	33.3	14.6–57.1
SATI > 57 cm^2^/m^2^	8	23.5

*IQR* Interquartile Range, *ECOG* Eastern Co-operative Oncology Group Performance Status, *ASA* American Society of Anesthesiologists risk score, *AACCI* Age Adjusted Charlson Comorbidity Index, *SMI* Skeletal Muscle Index, *MA* Muscle Attenuation, *HU* Hounsfield Unit, *VATI* Visceral Adipose Tissue Index, *SATI* Subcutaneous Adipose Tissue Index

### Surgical details and postoperative morbidity

Thirty-one patients (47.7%) underwent total exenteration, 20 (30.8%) anterior, and 14 (21.5%) posterior. Translevator and supralevator exenterations were performed in 41 (63.1%) and in 24 (36.9%) patients, respectively. In 7 patients (10.8%), pelvic side wall resection was performed en-bloc with the pelvic exenteration. Plastic reconstruction of the pelvic floor with or without reconstruction of the vulva, vagina, and/or perineum was performed in 32 patients (49.2%) and omental transposition in 42 (64.6%). Forty patients (61.5%) underwent 1 bowel anastomosis, and 4 (6.2%). Surgical resection with margins free of tumour (R0 resection) was achieved in 37 (56.9%) patients. In seven patients, detailed information on histological margins was missing. A median (IQR) duration of 610 min (475–740) and a median (IQR) number of four blood units (2–8) were required. Median (IQR) postoperative stay was 3 days (2–5) in the intensive care unit and 21 days (16–33) in the hospital. In 32 patients (49.2%), PN was given for a median (IQR) of 8 (6–12) days postoperatively. A total of 16 patients (24.6%) experienced Clavien–Dindo grade ≥ 3 postoperative morbidity within 60 days after pelvic exenteration. No 60 day post-operative mortality occurred.

### Body composition measurements

The median (IQR) BMI in our study population was 26.1 (23.6–32.9) kg/m^2^. Only two patients (3.1%) had a BMI < 18.5 and were therefore classified as underweight. Yet, data on BMI were missing in 19 patients. Seventeen patients (26.2%) had mild hypoalbuminemia with serum albumin levels between 2.5 and 3.49 g/dl. No patient had severe hypoalbuminemia.

The RMNST was completed prior to pelvic exenteration in 34 patients which revealed that 26 (76.5%) had low risk and 8 (23.5%) moderate risk for malnutrition. No patient had high risk for malnutrition prior to pelvic exenteration.

CT scans were performed in 54 patients prior to pelvic exenteration, and in 32 images cases were digitally archived and these were then subjected to sarcopenia analyses. We compared the group of patients used for sarcopenia analyses with the rest of patients and did not find any differences between the two groups with regard to patients’ age at time of surgery (*p* = *0.6*), ECOG performance status (*p* = *0.1*), type of pelvic exenteration (*p* = *0.2*), postoperative need for PN (*p* = *0.5*), severe 60 days postoperative morbidity (*p* = *0.5*), the occurrence of disease recurrence (*p* = *0.1*), or the patients’ status at time point of last follow-up (*p* = *0.4*). There were two patients who received chemotherapy within 60 days prior to the CT scan, one 10 days and the other one 33 days prior to CT scan. Both patients had high muscle attenuation. No other patients received chemotherapy or radiotherapy within 60 days prior to CT scan. Detailed clinical–pathological information in this group of patients is listed in supplementary Table 1.

**Fig. 1:**
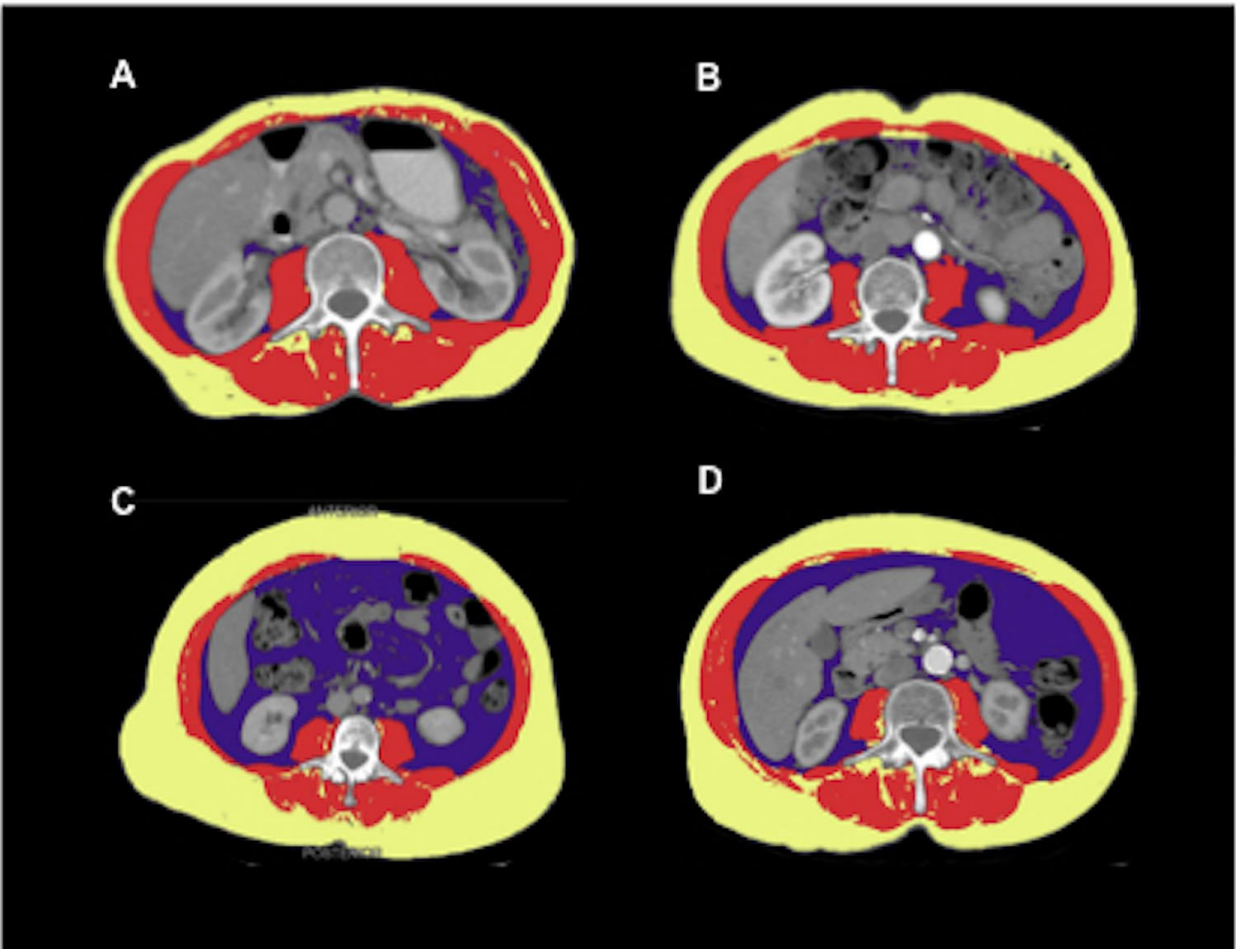
Examples for sarcopenia measurements in patients with high and low muscle attenuation. **A** and **B** depict examples for patients with normal muscle attenuation (MA): **A** 68 years, BMI 17.8 kg/m^2^, MA 42.3 Hounsfield units (HU); **B** 54 years, BMI 20.4 kg/m^2^, MA 41.8 HU; **C** and **D** depict examples for patients with low MA: **C** 69 years, BMI 32.9 kg/m^2^, MA 27.6 HU; **D** 73 years, BMI 24.8 kg/m^2^, MA 24.8 HU

Results of body composite measurements are shown in Table 1. Overweight and obese patients had a significantly lower MA compared to under- and normal-weight patients. The medians (IQR) of SMI, MA, VATI, and SATI for clinical risk groups are shown in Table 2. Examples for sarcopenia measurements in two patients with high MA, and in two patients with low MA are depicted in Fig. 1.

**Table 2:** Comparison of radiological body composition measurements between clinical risk groups (*n* = 32)

	Median SMI (IQR)	p*	Median MA (IQR)	p*	Median VATI (IQR)	p*	Median SATI (IQR)	p*
Age		0.3		0.9		0.6		0.008
< 55 years	42.9 (39.9–47.8)		41.8 (40.3–46.2)		93.5 (71.7–119.0)		21.5 (11.3–33.2)	
≥ 55 years	46.7 (42.0–51.5)		32.8 (27.6–41.8)		84.3 (57.7–135.0)		55.5 (33.3–77.7)	
BMI		–		0.04		–		–
< 25 kg/m^2^	–		41.8 (40.5–44.5)		–		–	
≥ 25 kg/m^2^	–		35.4 (32.1–43.8)		–		–	
Risk for malnutriation by RMNST		0.9		0.6		0.8		0.9
Low risk	44.8 (39.7–49.9)		40.9 (30.9–46.0)		82.4 (67.3–110.5)		31.2 (11.9–50.8)	
Moderate risk	43.3 (41.2–47.6)		34.3 (29.4–43.1)		94.2 (57.6–114.7)		34.9 (15.0–71.6)	

*SMI* Skeletal Muscle Index (cm^2^/m^2^), *MA* Muscle Attenuation (Hounsfield Units), *VATI* Visceral Adipose Tissue Index (cm^2^/m^2^), *SATI* Subcutaneous Adipose Tissue Index (cm^2^/m^2^), *BMI* Body Mass Index, *RMNST* Royal Marsden Nutritional Screening Tool

*Mann–Whitney-*U*-Test

We compared the medians of serum albumin between patients with SMI and MA below and above 41 cm^2^/m^2^ and 31 HU (i.e., 25th percentile), respectively. We found no difference of median serum albumin levels between the respective groups of SMI and MA (*p* = *0.5* and *p* = *0.4*, respectively). In logistic regression analyses, none of the body composition measurements were predictive of severe 60-day postoperative morbidity (i.e., Clavien–Dindo grade ≥ 3; see Table 3).

**Table 3:** Predictive value of clinico-laboratory parameters and body composition measurements for severe 60 days postoperative morbidity (Clavien Dindo ≥ grade 3)

	*p*	HR (95% CI)^a^
Age < 55 / ≥ 55 years	0.2	1.03 (0.9–1.1)
ECOG < 2 vs. ≥ 2	0.4	0.4 (0.06–2.9)
AACCI^b^	0.2	2.2 (0.6–9.0)
Radiotherapy in past (yes/no)	0.5	1.5 (0.4–5.7)
BMI ≥ 25 vs. < 25	0.2	2.5 (0.6–9.4)
Risk for malnutrition by RMNST^c^	0.6	0.7 (0.1–4.0)
Serum albumin	0.5	1.05 (0.9–1.2)
PN received post surgery	0.1	0.4 (0.1–1.4)
SMI < vs. ≥ 41 cm^2^/m^2^	0.7	0.7 (0.1–4.2)
MA < vs. ≥ 31 HU	0.9	1.02 (0.1–6.5)
VATI ≥ vs. < 120 cm^2^/m^2^	0.8	0.6 (0.1–3.2)
SATI ≥ vs. < 57 cm^2^/m^2^	–^d^	–

*HR* Hazard Ratio, *BMI* Body Mass Index, *PN* parenteral nutrition, *MA* Muscle Attenuation, *HU* Hounsfield Unit, *SMI* Skeletal Muscle Index, *SATI* Subcutaneous Adipose Tissue Index, *VATI* Visceral Adipose Tissue Index

^a^Logistic regression analyses

^b^Age Adjusted Charlson Comorbidity Index 0–4 vs. 5–7;

^c^Royal Marsden Nutritional Screening Tool: moderate vs. low risk for malnutrition

^d^Not enough events

### Association of nutritional factors and sarcopenia with survival outcomes

Median (IQR) follow-up for patients alive at last follow-up was 40.2 (21.6–76.7) months. Within this time, 35 patients (53.8%) died from their cancer, 5 patients (7.7%) from other causes. Forty patients (61.5%) had disease progression within a median (IQR) of 8.1 (5.7–14.6) months. Median (IQR) PFS and OS were 12 (6.9–28.8) and 20 (12–39.3) months, respectively. Median one-, two-, and three-year OS estimates (SE) were 80.3% (5.1), 50.5% (6.6), and 40.2% (6.7). Median PFS estimates (SE) at one, two, and three years were 59.1% (6.3), 41.8% (6.6), and 29.6% (6.6).

The results of uni- and multivariate survival analyses of the association of clinico-pathological, nutritional risk factors and body composition measurements with both PFS and OS are shown in Table 4.

**Table 4:** Association between clinico-pathological parameters, nutritional factors and body composition measurements and survival in patients undergoing pelvic exenteration for recurrent gynaecological malignancy

	Progression-free survival		Overall survival			
	Univariate		Univariate		Multivariate	
	*p*	HR (95% CI)*	*p*	HR (95% CI)^a^	*p*	HR (95% CI)^a^
Age < 55/ ≥ 55 years	0.5	0.8 (0.4–1.5)	0.5	0.8 (0.4–1.5)	–	–
ECOG ≥ 2 vs. < 2	0.7	1.2 (0.4–4.1)	0.8	1.1 (0.3–3.7)	–	–
AACCI^b^	0.7	0.9 (0.6–1.4)	0.5	0.9 (0.6–1.3)	–	–
Radiotherapy in the past	0.1	2.9 (0.7–11.9)	0.1	2.8 (0.7–12.3)	–	–
Histological Subtype^c^	0.4	0.9 (0.6–1.2)	0.1	0.8 (0.6–1.1)	–	–
Histological tumor size	0.4	1.01 (0.9–1.02)	0.5	1.0 (0.9–1.02)	–	–
Lymph nodes positive	0.3	0.6 (0.3–1.4)	0.8	0.9 (0.3–2.6)	–	–
Tumour resection margin positive	0.01	0.3 (0.1–0.8)	0.03	0.3 (0.1–0.9)	0.2	0.4 (0.1–1.7)
BMI ≥ 25 vs. < 25	0.6	1.3 (0.6–2.8)	0.9	0.9 (0.4–2.3)	–	–
Risk for malnutrition by RMNST^d^	0.009	3.7 (1.3–9.9)	0.006	0.2 (0.04–0.6)	0.03	0.13 (0.02–0.9)
Serum albumin	0.06	0.9 (0.9–1.0)	0.006	0.9 (0.8–0.9)	0.2	0.9 (0.7–1.1)
PN received post surgery	0.9	1.02 (0.5–1.9)	0.3	1.3 (0.7–2.5)	–	–
MA ≥ vs. < 31 HU	0.5	1.5 (0.5–4.7)	0.04	0.3 (0.1–1.9)	0.02	0.1 (0.01–1.7)
SMI < vs. ≥ 41 cm^2^/m^2^	0.09	0.4 (0.1–1.1)	0.2	0.4 (0.1–1.4)	–	–
SATI ≥ vs. < 57 cm^2^/m^2^	0.8	1.1 (0.4–3.6)	0.7	1.2 (0.4–4.0)	–	–
VATI ≥ vs. < 120 cm^2^/m^2^	0.5	1.5 (0.5–4.8)	0.5	1.5 (0.5–5.1)	–	–

*HR* Hazard Ratio, *CI* Confidence Interval, *BMI* Body Mass Index, *PN* parenteral nutrition, *MA* Muscle Attenuation, *HU* Hounsfield Unit, *SMI* Skeletal Muscle Index, *SATI* Subcutaneous Adipose Tissue Index, *VATI* Visceral Adipose Tissue Index

^a^Cox regression analysis

^b^Age Adjusted Charlson Comorbidity Index 0–4 vs. 5–7

^c^Histological subtype of primary cancer: serous/mixed serous-mucinous vs. endometrioid vs. SCC/adenosquamous vs. leiomyosarcoma/endometrial stromal sarcoma

^d^Royal Marsden Nutritional Screening Tool (moderate vs. low risk for malnutrition)

**Fig. 2:**
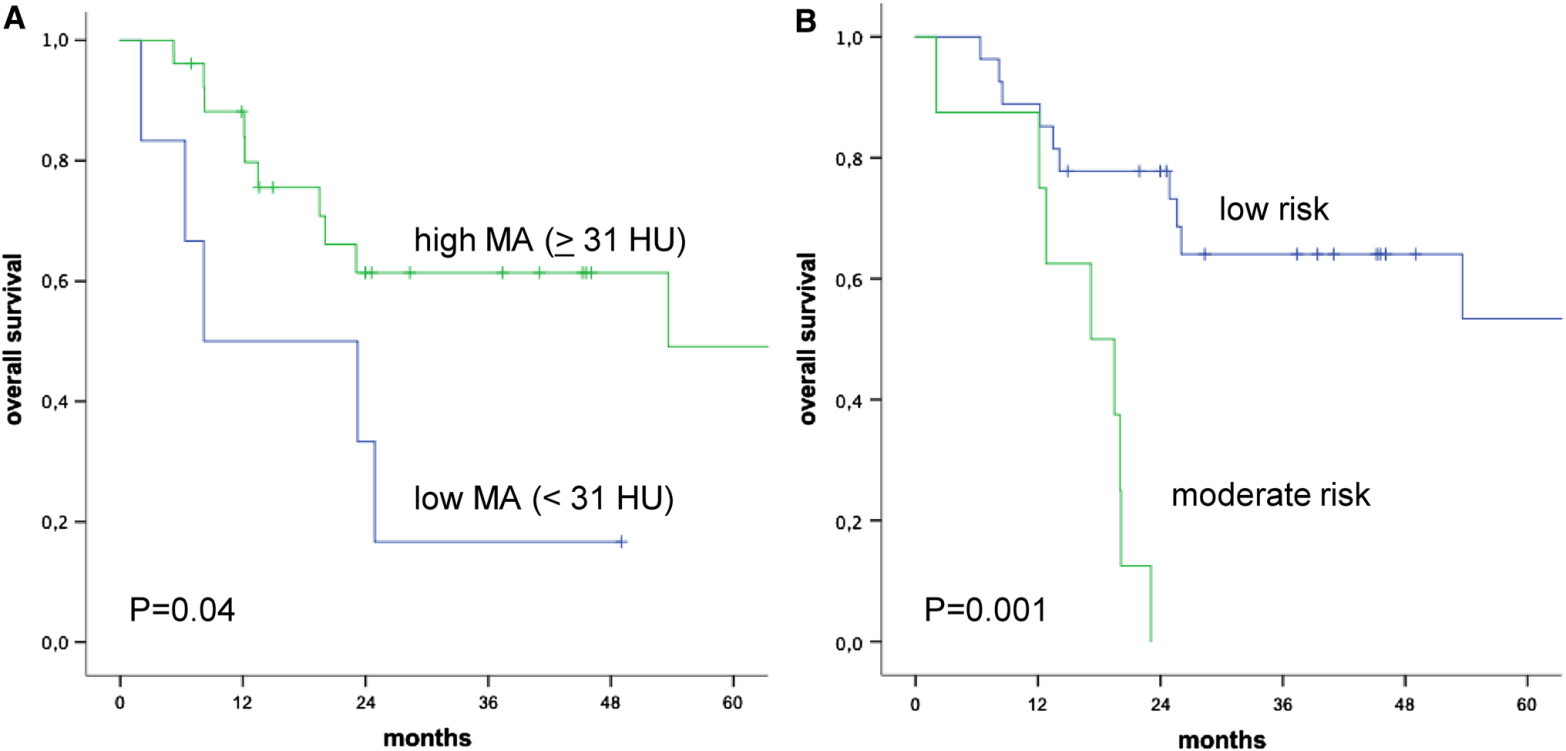
Kaplan–Meier curves for overall survival. Kaplan–Meier curves depicting overall survival in patients treated with pelvic exenteration for gynaecological malignancies, stratified according to (**A**) muscle attenuation (MA) and **B** risk for malnutrition according to the Royal Marsden Nutrition Screening Tool criteria

Figure 2 shows Kaplan–Meier curves depicting the association of MA and RMNST risk groups with OS. We found moderate risk for malnutrition based on the RMNST to be the only factor associated with shorter PFS in univariate analysis. In addition to risk for malnutrition based on the RMNST, serum albumin and a low MA (< 31 HU) were associated with shorter OS in univariate analysis.

In a multivariate analysis that adjusted for the effects of all factors associated with survival in univariate analyses, MA and moderate risk for malnutrition based on the RMNST remained independently associated with shorter OS.

## Discussion

Preoperative prediction of postoperative morbidity and survival in patients undergoing major surgery, including pelvic exenteration, is essential for patient selection to assure optimal outcomes. To our knowledge, this is the first study to determine if malnutrition and sarcopenia impact on postoperative morbidity and survival in patients with recurrent gynaecological malignancies treated with pelvic exenteration. Our results demonstrate that low MA and moderate risk for malnutrition, based on the RMNST, are independent prognostic factors for survival in these patients.

The current eligibility for selection of patients with a recurrent gynaecological malignancy to undergo pelvic exenteration is fundamentally based on the pre-operative assessment that the disease is limited to the pelvis and can therefore be completely removed by surgical resection. However, only 20–50% of patients, depending on the type of primary cancer site, reportedly have a substantial benefit with long-term survival improvement [10]. Sarcopenia, a key component of cachexia, has been shown to be associated with decreased survival in various malignancies [12–16]. In ovarian cancer, few studies have investigated the effects of sarcopenia and found that low MA and SMI were associated with shorter OS [15, 16, 30].

We found that low MA was an independent predictor for shorter OS in patients treated with pelvic exenteration for recurrent gynaecological malignancies. Current research suggests that loss of muscle quality compared to loss of muscle mass has a greater negative impact on muscle function [20]. Various molecular mechanisms leading to energy-wasting and loss of myofibrillar proteins in skeletal muscle cells, including the impaired function of skeletal muscle mitochondria and the secretion of inflammatory cytokines by immune or tumour cells, have been shown to result from the presence of malignancy [19].

The median MA and SMI of our study population were similar to results reported for advanced ovarian cancer patients, but substantially higher than in patients with pancreatic cancer [15, 31]. Taking into account, that all patients in the present study had recurrent, often heavily pretreated disease, the comparably favourable sarcopenia measurements may attest to the patient selection process for pelvic exenteration at our institution.

Another interesting finding was the correlation between MA and the patient’s BMI. In contrast to historical assumptions of cachexia (and hence sarcopenia) affecting only patients with low BMI, our results suggest a higher risk for sarcopenia in overweight and obese patients. We believe this to be an important clinical observation. These findings correlate with previously reported associations between obesity and sarcopenia and underline the risk of underdiagnosing sarcopenia when relying on BMI or overall weight loss only [12, 32].

In addition to radiological sarcopenia measurements, we evaluated the accuracy of the RMNST, a validated questionnaire, used as a screening tool for nutritional status [28]. In obese patients the questionnaire may be detecting weight loss—and it has been reported that even on those patients with a high BMI, weight loss can be a risk factor for poorer outcome [11]. We demonstrated that moderate malnutrition was an independent marker for both PFS and OS in our study group of patients. Interestingly, none of the patients had risk for severe malnutrition according to the RMNST. This is not unexpected as only patients estimated to be fit enough to undergo pelvic exenteration were selected for surgery.

Interestingly, neither radiological body composite measurements nor the risk of malnutrition according to the RMNST were associated with postoperative morbidity. In contrast to our results, sarcopenia was reported to be a risk factor for postoperative morbidity after pancreaticoduodenectomy and after gastrectomy [22, 23, 31, 33, 34]. The different outcomes of our study might be either due to the relatively small sample size or to a patient selection bias. Surgical outcome in our study in terms of median intraoperative blood loss, length of hospital stay, and postoperative morbidity were similar to those previously reported [3–6, 9, 10]. In coherence with other studies, R0 resection had a strong impact on progression free and overall survival [35]. However, when performing multivariable analysis only MA and risk of malnutrition according to the RMNST were independently associated with overall survival. Considering the small and heterogenous sample size this finding has to be interpreted with caution.

The main limitations of our study are its retrospective design and the relatively small sample size. Patients were only included if CT scans prior to pelvic exenteration were available for analysis, potentially causing a selection bias. A cohort of 32 patients is too low to draw a precise conclusion. However, taking into consideration of the scarcity of patients, who underwent pelvic exenteration for recurrent disease, it will be difficult to perform similar analyses within a bigger and more homogenous patients’ cohort. Furthermore, there is not yet a standardized threshold value for SMI nor precisely defined threshold values for SMI and MA. As our study was retrospective, we could not combine SMI or MA with a functional muscle assessment. Furthermore, multiple statistical tests were performed, increasing the risk of committing type 1 errors.

We hope our results will be regarded as hypothesis generating. Further prospective studies, which should include functional muscle assessment are needed to validate these results in a larger cohort of patients undergoing pelvic exenteration. 

## Conclusions

In summary, our results demonstrate, for the first time, that reduced MA and malnutrition as assessed by the RMNST questionnaire are associated with reduced survival in patients treated with pelvic exenteration for recurrent gynaecological malignancies. As CT scans are performed routinely prior to pelvic exenteration, the measurement of MA could easily be incorporated in the pre-operative assessment of these patients. Especially for the planning of palliative exenteration, this could add value to appropriate patient selection. If our results are validated in larger controlled studies, these parameters could be used in clinical decision making, case selection and counselling of patients.

## Supplementary Information

Below is the link to the electronic supplementary material.

Supplementary file1 S 1. Flow chart depicting the selection process of patients for analyses of the present study. (JPG 924 KB)

Supplementary file2 (DOCX 16 KB)
