# Supplementary figures and images for: The impact of nutritional risk factors and sarcopenia on survival in patients treated with pelvic exenteration for recurrent gynaecological malignancy: a retrospective cohort study
Source: Arch Gynecol Obstet. 2021 Nov 3;305(5):1343–52. doi: 10.1007/s00404-021-06273-7 (PMC9013326; doi:10.1007/s00404-021-06273-7)

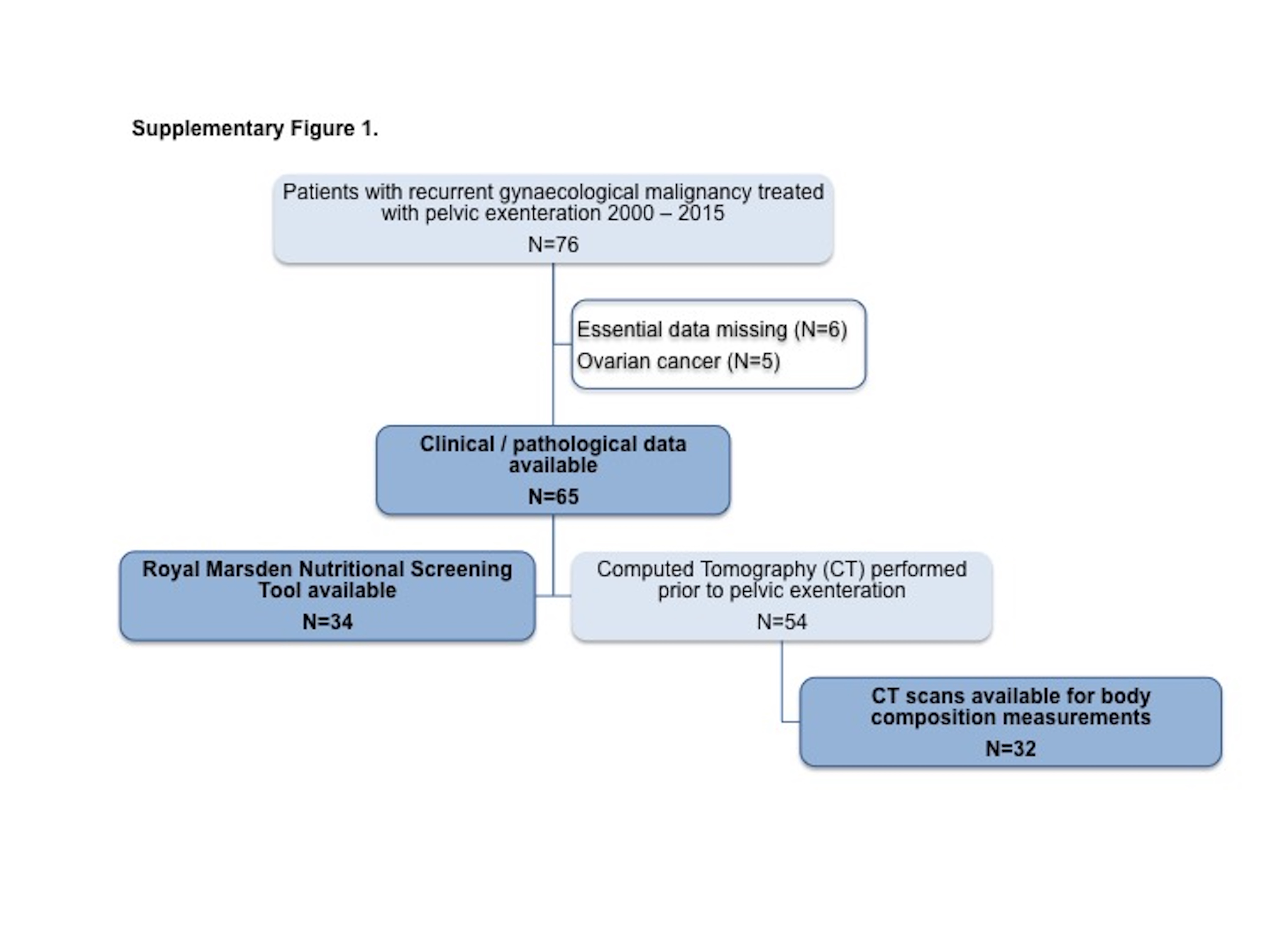

Supplement: Supplementary file 1 — Supplementary file1 S 1. Flow chart depicting the selection process of patients for analyses of the present study. (JPG 924 KB) [file 404_2021_6273_MOESM1_ESM.jpg]
