# Supplementary material for: The impact of nutritional risk factors and sarcopenia on survival in patients treated with pelvic exenteration for recurrent gynaecological malignancy: a retrospective cohort study
Source: Arch Gynecol Obstet. 2021 Nov 3;305(5):1343–52. doi: 10.1007/s00404-021-06273-7 (PMC9013326; doi:10.1007/s00404-021-06273-7)
Supplement: Supplementary file 2 — Supplementary file2 (DOCX 16 KB) [file 404_2021_6273_MOESM2_ESM.docx]

**Supplementary Table 1.** Patients’ characteristics compared in cohorts with low and high muscle attenuation (n=32).

|  | **Low MA (n=7)** | **High MA (n=25)** | **p-value** |
| --- | --- | --- | --- |
|  | **N (%)** | **N (%)** |  |
| **Age in years (median, IQR)** | 62 (59-69) | 52 (44-64) | 0.03* |
| **Recurrent / persistent cancer of** |  |  | 0.3° |
| vulva / vagina | 4 (57.2) | 9 (36) |  |
| cervix | 1 (14.2) | 11 (44) |  |
| uterus | 2 (28.6) | 5 (20) |  |
| **Histology** |  |  | 0.6° |
| adenocarcinoma | 3 (42.9) | 8 (32) |  |
| squamous cell carcinoma | 3 (42.9) | 14 (56) |  |
| clear cell carcinoma | 0 | 1 (4) |  |
| mixed mullerian tumour | 0 | 1 (4) |  |
| sarcoma | 1 (14.2) | 1 (4) |  |
| **Radiotherapy in the past** | 5 (71.4) | 22 (91.7) | 0.1° |
| **AACCI** |  |  | 0.4° |
| 2 | 0 | 8 (32) |  |
| 3 | 2 (28.6) | 6 (24) |  |
| 4 | 2 (28.6) | 2 (8) |  |
| 5 | 2 (28.6) | 4 (16) |  |
| 6 | 1 (14.2) | 3 (12) |  |
| 7 | 0 | 1 (4) |  |
| missing | - | 1 (4) |  |
| **Risk for malnutrition by RMNST** |  |  | 0.2° |
| low | 4 (57.2) | 14 (56) |  |
| intermediate | 1 (14.2) | 4 (16) |  |
| high | 0 | 0 |  |
| missing | 2 (28.6) | 7 |  |
| **BMI** |  |  | 0.8° |
| Underweight (BMI <=18) | 0 | 1 (4) |  |
| Normal weight (BMI >18 and <=25) | 2 (28.6) | 11 (44) |  |
| Overweight (BMI >25) | 3 (42.8) | 12 (48) |  |
| Missing | 2 (28.6) | 1 (4) |  |
| **Albumin (median, IQR)** | 36 (34-42) | 38 (35-42) | 0.2* |
| **Positive tumour resection margins** | 3 (42.8) | 7 (28) | 0.4° |
| **Lymph node involvement** | 1 (14.2) | 2 (8) |  |
| **Recurrence** | 4 (57.1) | 13 (52) | 0.8° |
| **Status at last observation** |  |  | 0.2° |
| Alive without disease | 1 (14.3) | 11 (44) |  |
| Stable disease | 0 | 2 (8) |  |
| Progressive disease | 0 | 1 (4) |  |
| Cancer-related death | 4 (57.1) | 10 (40) |  |
| Non-cancer related death | 2 (28.6) | 1 (4) |  |

MA=Muscle Attenuation; IQR=interquartile range; AACCI=Age Adjusted Charlson Comorbidity Index; RMNST=Royal Marsden Nutritional Screening Tool; BMI=Body Mass Index; *Mann-Whitney-U-Test; °Chi-square test
